# Discovery of small molecules against porcine reproductive and respiratory syndrome virus replication by targeting NendoU activity

**DOI:** 10.1128/jvi.02034-24

**Published:** 2024-12-31

**Authors:** Jiaqi Zhu, Yunqiang Lai, Mengqi Cheng, Radha Charan Dash, Shuangshuang Guo, Jintong Guo, Yue Su, Andrew Wolek, Brianna Issacs, Zhenming Liu, Qi Li, Neha Mishra, Antonio Garmendia, M. Kyle Hadden, X. Cindy Tian, Xin He, Young Tang

**Affiliations:** 1 Department of Animal Science, Institute for Systems Genomics, University of Connecticut 703792 , Storrs, Connecticut, USA; 2 Shaanxi Centre of Stem Cells Engineering & Technology, Key Laboratory of Livestock Biology, Engineering Research Center of Efficient New Vaccines for Animals, Ministry of Education and Universities of Shaanxi Province, College of Veterinary Medicine, Northwest A&F University 12469 , Yangling, Shaanxi, China; 3 Department of Pharmaceutical Sciences, University of Connecticut 242849 , Storrs, Connecticut, USA; 4 State Key Laboratory of Natural and Biomimetic Drugs, School of Pharmaceutical Sciences, Peking University 208324 , Beijing, China; 5 Department of Pathobiology and Veterinary Sciences, University of Connecticut 7712 , Storrs, Connecticut, USA; University of Michigan Medical School, Ann Arbor, Michigan, USA

**Keywords:** PRRSV, NendoU, nidovirus, antiviral agents

## Abstract

Porcine reproductive and respiratory syndrome (PRRS) remains a major threat to animal health and causes substantial economic losses worldwide. The nonstructural protein 11 (NSP11) of the causative agent, PRRS virus (PRRSV), contains a highly conserved nidoviral uridylate-specific endoribonuclease (NendoU) domain essential for viral replication and immune evasion. Targeting NSP11 offers a novel approach to antiviral intervention. Through *in silico* virtual screening followed by a fluorescence resonance energy transfer assay , we identified A8-A2 as a promising candidate that effectively inhibits NendoU activity. Molecular docking and mutational analysis revealed that A8-A2 and its analogs target the key catalytic residues His144 and Thr217 of NSP11, located within the NendoU enzyme activity loop and pocket region, respectively. A8-A2 demonstrated dose-dependent inhibition of PRRSV replication in porcine alveolar macrophages. Notably, the NendoU is conserved across PRRSV strains and other Nidoviruses, and A8-A2 exhibited antiviral activity against both type I and type II PRRSV strains, as well as the infectious bronchitis virus, a coronavirus in the order *Nidovirales*. Further investigations revealed that A8-A2 impedes viral replication early in infection and reverses NSP11-mediated suppression of Poly(I:C)-induced interferon production. However, this effect occurs independently of mRNA splicing inhibition. These findings indicate that A8-A2 could act as an effective antiviral agent against infections caused by diverse PRRSV strains and may serve as a broad-spectrum agent for other Nidoviruses.

**IMPORTANCE:**

Porcine reproductive and respiratory syndrome virus (PRRSV) causes significant economic losses in the pig industry, and vaccination is the principal method to prevent this viral infection currently. However, vaccination often fails to provide protection against heterologous strains, highlighting the need for alternative strategies for broad protection. The nidoviral uridylate-specific endoribonuclease (NendoU) domain plays a crucial role in viral replication and evasion of host immune responses. In this study, we identified a group of new compounds with similar chemical structures that could interfere with NendoU enzyme activity. Among these compounds, A8-A2 significantly inhibited PRRSV replication in host cells with minimal cytotoxicity. Our findings provide a new direction for developing potent antiviral compounds that can offer broad protection against different PRRSV strains, thereby mitigating their impact on pig health and benefiting the husbandry industry.

## INTRODUCTION

Porcine reproductive and respiratory syndrome (PRRS) is one of the most economically significant swine diseases worldwide, with an estimated annual cost of at least $600 million in the United States (1, 2). The causative agent, PRRS virus (PRRSV) is an enveloped, positive-sense, single-stranded RNA virus belonging to the order *Nidovirales*, family *Arteriviridae* (3, 4). PRRSV can be classified into two genotypes: type I (European) and type II (North American), with VR-2332 (VR) and Lelystad (LY) as the prototype strains, respectively (5, 6). Both genotypes can cause reproductive failure in sows and respiratory disease in piglets; however, they share only 30–50% amino acid identity, creating a significant challenge for developing cross-protective antiviral agents (7, 8).

The PRRSV genome, approximately 15 kb in length, encodes at least nine open reading frames (ORFs). ORF1a and ORF1b constitute three-quarters of the genome, encoding two large polyproteins that undergo proteolytic processing to yield at least 12 nonstructural proteins (NSPs) essential for viral replication (9). Among these, nonstructural protein 11 (NSP11) plays a pivotal role in PRRSV pathogenesis. NSP11 contains a nidoviral uridylate-specific endoribonuclease (NendoU) domain, which is characteristic of nidoviruses and shares structural similarities with the endoribonuclease XendoU from *Xenopus laevis*. This NendoU domain is a defining genetic feature of nidoviruses, distinguishing them from other RNA viruses (10, 11).

The NendoU activity is essential for nidoviral replication. Overexpression of NSP11 enhances PRRSV infectivity, while its inhibition restricts viral replication (12, 13). Key catalytic residues, such as histidines in the enzyme activity loop, and lysine or threonine in the supporting loop, are crucial for maintaining NendoU activity (14, 15). PRRSV with mutations in the NSP11 catalytic sites lose infectivity and fail to produce progeny (16), indicating a direct contribution of NSP11 to viral replication. Additionally, deletion of the NendoU domain or parts of its structure in equine arteritis virus, another arterivirus, is lethal, suggesting that an intact NendoU domain is critical for arterivirus replication (17). Mutations at the catalytic site also substantially impair subgenomic RNA synthesis and progeny production, even in interferon (IFN)-deficient cells (17).

NSP11 also plays an important role in immune evasion by interfering with the type I IFN (IFN-I) signaling pathway. NSP11 suppresses Poly(I:C) (a synthetic dsRNA analog)-induced IFN-β production, an effect that is abolished by mutations in the NendoU catalytic residues (His-129, His-144, and Lys-170) (18). NSP11 disrupts various steps in IFN production. For example, it downregulates IFN-β, IRF3, and NF-κB expression by interfering with mitochondrial antiviral-signaling protein (MAVS) and RIG-I (16, 19). Moreover, NSP11 recruits ovarian tumor domain deubiquitinase with linear linkage specificity (OTULIN), a deubiquitinase that removes linear ubiquitination from NF-κB essential modulator (NEMO), leading to suppressed IFN-I responses (20). Additionally, the N-terminal domain (NTD) of NSP11 promotes the degradation of STAT2, further disrupting the IFN pathway (21).

The multifunctional roles of NendoU in promoting PRRSV replication and evading host immunity underscore its importance in PRRSV pathogenesis. Thus, small molecules targeting NendoU have the potential to inhibit the PRRSV replication. Currently, no specific inhibitors of NSP11 in arteriviruses have been reported. Our study introduces a novel approach for antiviral developing, aiming to control PRRSV infection through targeted inhibition of NSP11.

## RESULTS

### Screening for inhibitors of PPRSV NSP11 NendoU activity

**Fig 1:**
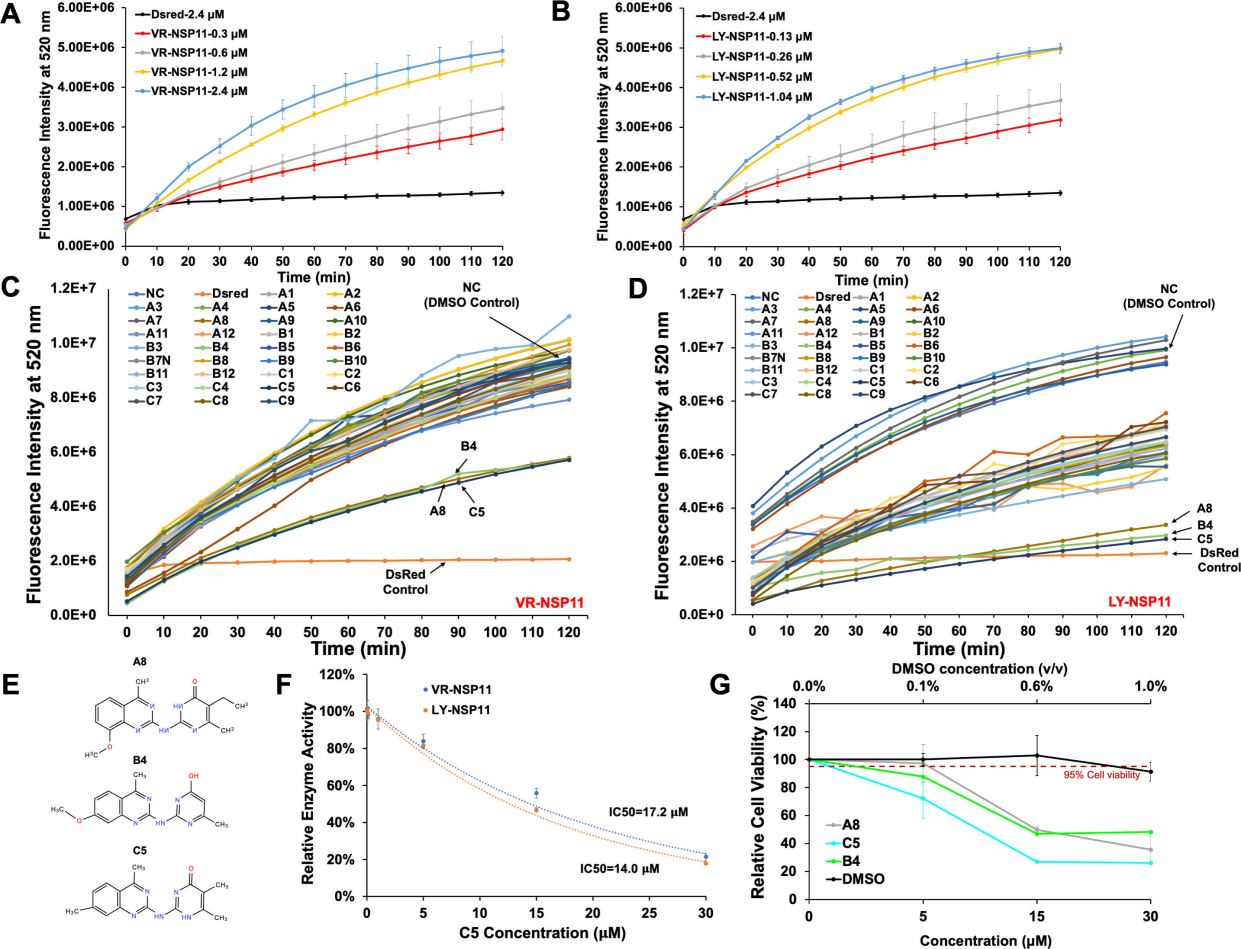
Screening for inhibitors of PRRSV NSP11 NendoU activity. NendoU activity analysis of VR-NSP11 (A) and LY-NSP11 (B). Different concentrations of VR-NSP11 or LY-NSP11 protein were incubated with R23.1 oligos to measure the endoribonuclease activity every 20 min. DsRed: negative protein control. DsRed were incubated with R23.1 oligo. Values represent mean ± SD, *n* = 3. NendoU activity inhibition analysis for VR-NSP11 (C) and LY-NSP11 (D). 1.2 µM VR-NSP11 and 0.52 µM LY-NSP11 were incubated with R23.1 oligo and each of the 33 compounds at 15 µM level. NC: negative vehicle control with NSP11 and R23.1 oligo mixed with DMSO; DsRed: negative protein control with DsRed and R23.1 oligo mixed with DMSO. Values represent mean ± SD, *n* = 3. (E) Chemical structures of compounds A8, B4, and C5. (F) Dosage-dependent inhibitory activity of compound C5 to NSP11 at 2 h. Values represent mean ± SD, *n* = 3. (G) Cytotoxicity analysis of A8, B4, and C5. Compounds were incubated with PAMs for 48 h, with DMSO as the vehicle control. The red dashed line indicates 95% relative viability. The top *x*-axis illustrates DMSO concentration, expressed as the ratio of DMSO volume to medium volume (vol/vol). The bottom *x*-axis denotes compound concentrations (µM). Values represent mean ± SD, *n* = 3.

We initially established a NendoU assay to identify compounds capable of inhibiting the endoribonuclease activity of PRRSV NSP11. We expressed and purified NSP11 proteins from type II PRRSV VR-2332 strain (VR) and type I PRRSV Lelystad strain (LY) (VR-NSP11 and LY-NSP11), both fused with an N-terminal histidine and asparagine (HN) tag (Fig. S1A). Both VR- and LY-NSP11 proteins exhibited dose-dependent endoribonuclease activity when incubated with R23.1, a 23-mer RNA oligo labeled with fluorescein (5′ 6-FAM) at the 5′ end and Black Hole Quencher (BHQ-1) at the 3′ end (Fig. 1A and B). Simultaneously, we performed a docking-based virtual screen of approximately 155,000 small molecules to identify those with the potential to bind to the known NSP11 catalytic sites. This screen identified 33 candidates (A1-C9), which were further evaluated at 15 µM in our validated NendoU assay (Table S1). Of these, three compounds—A8, B4, and C5—exhibited significant inhibition of both VR- and LY-NSP11 enzyme activities (Fig. 1C and D). These compounds, all substituted quinazolines, share very similar chemical structures (Fig. 1E). Compound C5 was further evaluated and demonstrated concentration-dependent inhibition of LY- and VR-NSP11, with 50% effective inhibitory concentration (IC_50_) values of 14.0 and 17.2 µM, respectively, based on fluorescence intensity measurements at 2 h (Fig. 1F). PRRSV primarily targets cells of the monocyte/macrophage lineage, particularly porcine alveolar macrophages (PAMs) in pig lungs (22, 23). We then assessed the cytotoxicity of these active compounds on PAMs and found, unexpectedly, that all three significantly reduced the cell viability to below 50% at a concentration of 15 µM, comparable to the IC_50_ value of C5, indicating low selectivity (Fig. 1G).

### Assessment of the effect and cytotoxicity of NSP11 inhibitor analogs

**Fig 2:**
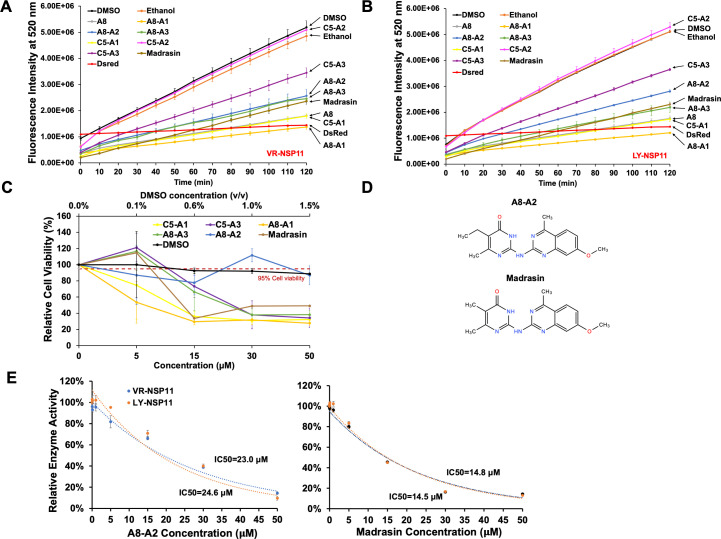
Assessment of the cytotoxicity of NSP11 inhibitor analogs. NendoU activity inhibition analysis for VR-NSP11 (A) and LY-NSP11 (B). VR-NSP11 and LY-NSP11 were incubated with R23.1 oligo and each of the seven additional analogs at 15 µM level. DMSO or ethanol: negative vehicle control with NSP11 and R23.1 oligo mixed with DMSO or ethanol; DsRed: negative protein control with DsRed and R23.1 oligo mixed with DMSO. Values represent mean ± SD, *n* = 3. (**C**) MTT analysis of the six additional analogs incubated with PAMs for 48 h, with DMSO as the vehicle control. The red dashed line indicates 95% relative viability. The top *x*-axis illustrates DMSO concentration, expressed as the ratio of DMSO volume to medium volume (vol/vol). The bottom *x*-axis denotes compound concentrations (µM). Values represent mean ± SD, *n* = 3. (**D**) Chemical structures of compounds A8-A2 and Madrasin. (**E**) Dosage-dependent inhibitory activity of compound A8-A2 (left) and Madrasin (right) to VR/LY-NSP11. Values represent mean ± SD, *n* = 3.

To identify alternative compounds with similar effects but lower cytotoxicity, we examined six additional analogs (A8-A1, A8-A2, A8-A3, C5-A1, C5-A2, and C5-A3) from ChemBridge Corporation, as well as Madrasin, which was reported as a pre-mRNA splicing inhibitor to halt spliceosome assembly (24). These analogs share structural similarities with either A8 or C5 (Table S1). In the NendoU assay, all compounds except C5-A2 showed inhibitory effects on both VR- and LY-NSP11 activities at a concentration of 15 µM (Fig. 2A and B). Notably, A8-A2 demonstrated no significant reduction in PAMs viability at concentrations up to 50 µM, indicating a 50% cytotoxic concentration (CC_50_) exceeding 50 µM, which makes it a strong candidate for further development. In contrast, the cell viability drops quickly with the treatment of other analogs to around 30% at 50 µM (Fig. 2C). Structurally, A8-A2 is similar to both A8 and Madrasin (Fig. 1E and 2D) and achieved IC_50_ values of 23.0 and 24.6 µM for LY- and VR-NSP11, respectively, in the NendoU assay at 2 h. Although these IC_50_ values are higher than those of C5 and Madrasin (Fig. 1F and 2E), they confirm that A8-A2 effectively inhibits NSP11 activity while maintaining a favorable safety profile.

### Analysis of binding interactions between NSP11 and its inhibitors

**Fig 3:**
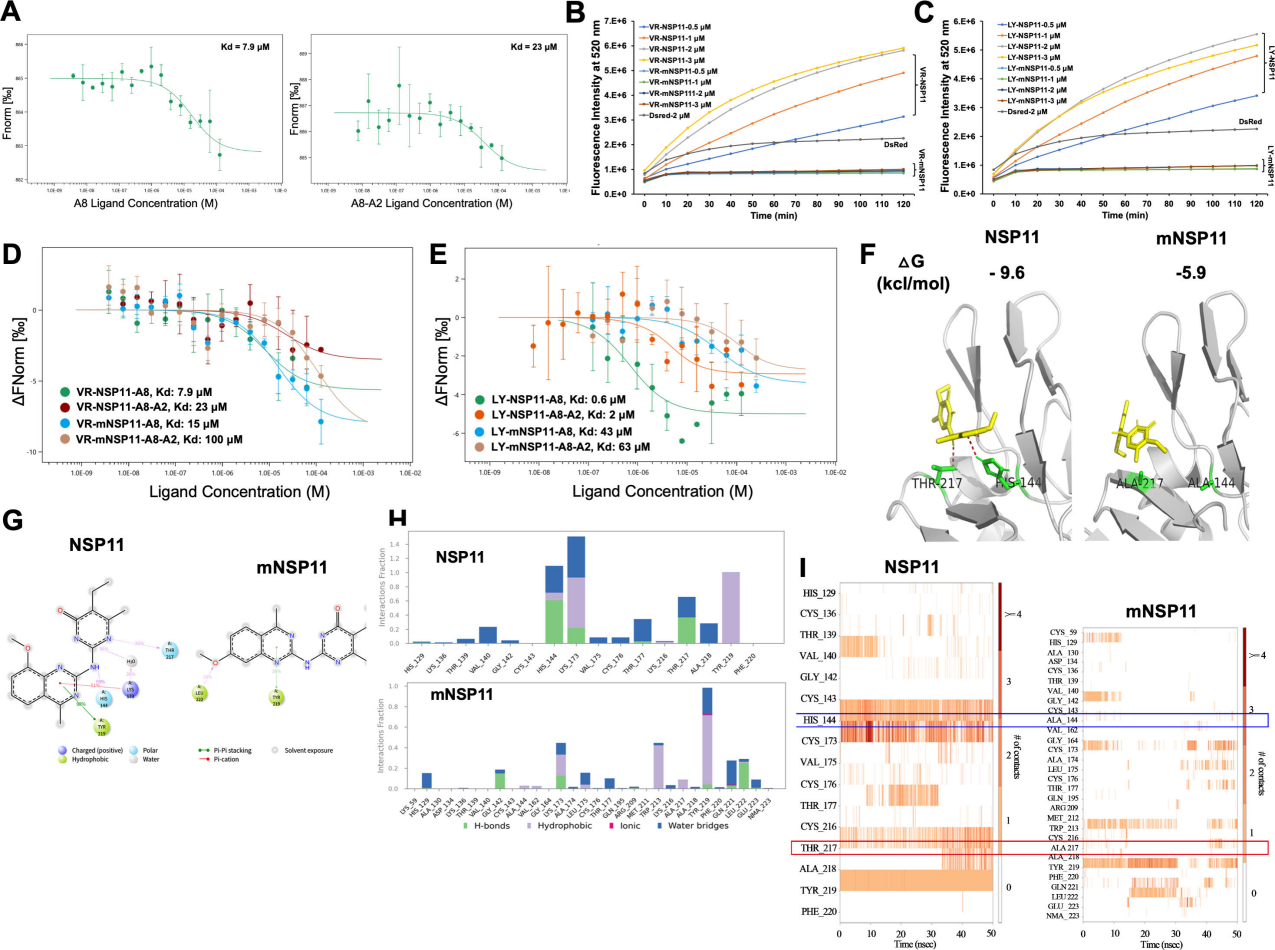
Binding interaction between NSP11 and its inhibitors. (**A**) MST analysis of NHS-labeled VR-NSP11 thermal dynamic association with ligands A8 (left) and A8-A2 (right). Values represent mean ± SD, *n* = 3. (**B**) NendoU activity analysis with different concentrations of wild-type VR-NSP11 and VR-mNSP11. DsRed protein was used as the negative protein control. Values represent mean ± SD, *n* = 3. (**C**) NendoU assay analysis with different concentrations of wild-type LY-NSP11 and LY-mNSP11. DsRed protein was used as the negative protein control. Values represent mean ± SD, *n* = 3. (**D**) MST analysis of NHS-labeed VR-NSP11 and VR-mutated NSP11 (VR-mNSP11) protein thermal dynamic association with ligand A8 or A8-A2. Values represent mean ± SD, *n* = 3. (**E**) MST analysis of His-tag labeled LY-NSP11 and LY-mNSP11 protein thermal dynamic association with ligand A8 or A8-A2. Values represent mean ± SD, *n* = 3. (**F**) Molecular docking analysis illustrating the potential interactions between compound A8-A2 with the catalytic region of wild-type NSP11 and mNSP11. (**G**) Molecular dynamics simulation highlighting the hydrogen bonds formed between A8-A2 and both wild-type and mNSP11. (**H**) Interaction fractions of molecular bonds between A8-A2 and both wild-type and mNSP11. (**I**) Phase diagram from molecular dynamics simulation showing the hydrogen bonds between A8-A2 and both wild-type and mNSP11.

To investigate whether the positive hits inhibit NSP11 enzyme activity through direct intermolecular interaction, we performed microscale thermophoresis (MST) analysis (25). RED-N-Hydroxysuccinimide (NHS)-ester labeled NSP11, targeting lysine residues, was incubated with either compound A8 or A8-A2. The MST analysis confirmed a direct interaction between VR-NSP11 and both compounds (Fig. 3A). Molecular docking further revealed that compounds A8, A8-A2, and C5 have strong binding affinity for the catalytic groove of NSP11, specifically forming hydrogen bonds with residues H144 in the catalytic active loop and T217 in the catalytic pocket (Fig. S2 and B). To evaluate the significance of these potential hydrogen bonds for ligand-NSP11 binding, we expressed and purified mutant VR- and LY-NSP11 proteins (mNSP11) with H144A and T217A substitutions (Fig. S1A and B). Both mutants, as expected, showed complete loss of NendoU activity (Fig. 3B and C). MST analysis indicated that the dissociation constant (Kd) for compounds A8 and A8-A2 with VR-mNSP11 increased by 1.9- and 4.3-fold, respectively, compared to the wild type (Fig. 3D). Given that RED-NHS-ester may affect binding by targeting multiple catalytic lysine residues, we then used RED-tris-NTA to label the His-tag of the recombinant proteins for MST analysis. This approach showed a greater Kd shift in LY-mNSP11 compared to the wild type, with an increase of 71.7- and 31.5-fold for A8 and A8-A2, respectively (Fig. 3E).

To further explore hydrogen bond formation between NSP11 and A8-A2, additional molecular docking analysis confirms that A8-A2 forms hydrogen bonds with NSP11 at His144 and Thr217 which are disrupted in mNSP11. The binding energy change (Δ*G*) increased from −9.6 kcal/mol in wild-type NSP11 to −5.9 kcal/mol in mNSP11, indicating reduced affinity of A8-A2 binding upon mutation (Fig. 3F). Molecular dynamics simulations supported these findings, showing that A8-A2 primarily interacts through hydrogen bonds with His144 and Thr217 in NSP11 and through hydrophobic interactions with Lys173 and Tyr219, alongside water-bridged contacts with His144, Lys173, Thr177, Thr217, and Ala218. These interactions were markedly altered in mNSP11 (Fig. 3G and H). Furthermore, molecular dynamics simulations demonstrated that the hydrogen bonds of A8-A2 with His144 and Thr217 in NSP11 were fully eliminated in mNSP11 (Fig. 3I). These findings collectively underscore the essential role of hydrogen bonds at His144 and Thr217 for effective ligand-protein interaction in NSP11.

### Validation of the NSP11 inhibitor efficacy against PRRSV infection

**Fig 4:**
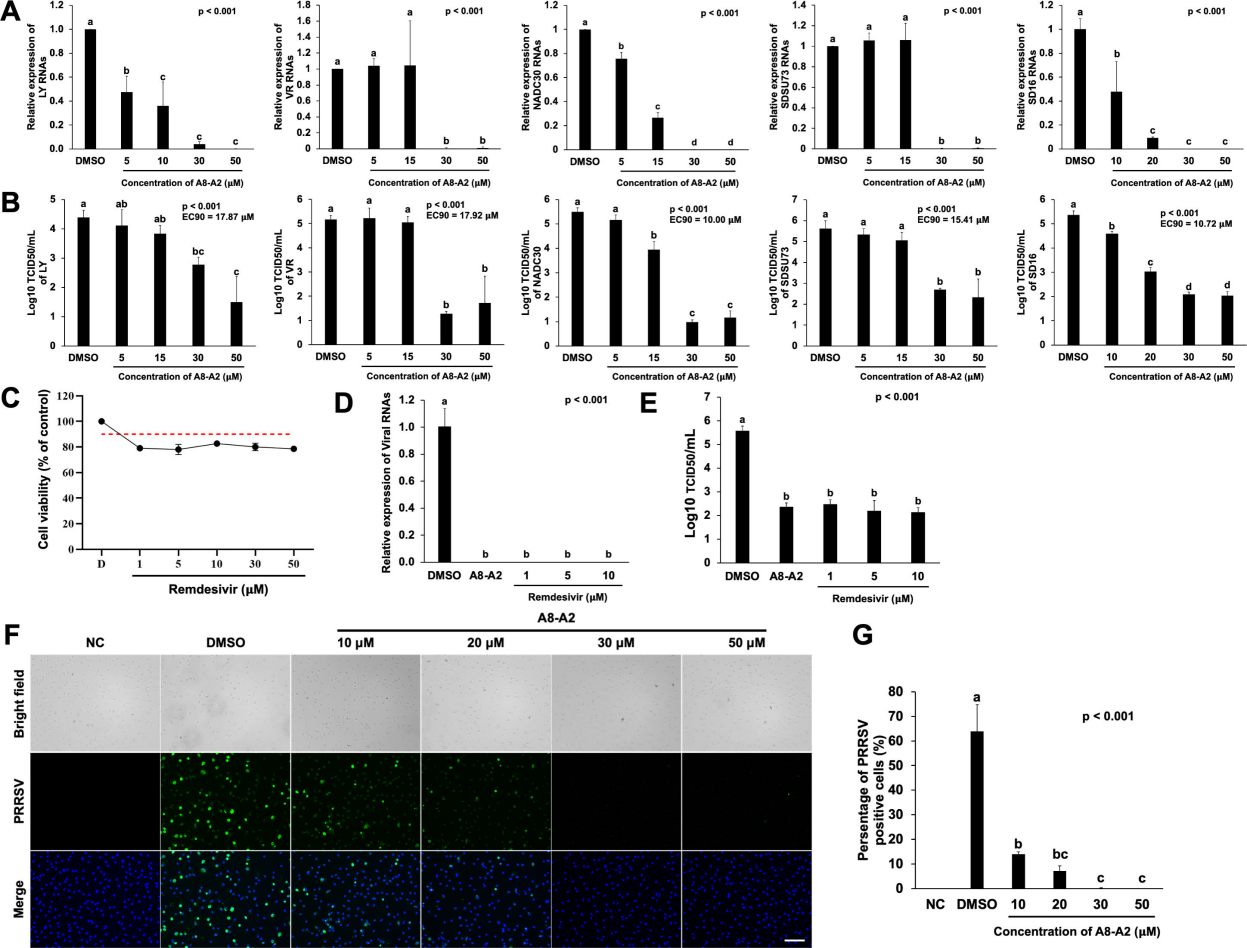
Validation of the NSP11 inhibitor efficacy against PRRSV infection. (**A**) qRT-PCR analysis of LY, VR, NADC30, SDSU73, and SD16 RNA in PAMs treated with different concentrations of A8-A2. DMSO was used as negative controls. Values represent mean ± SD, *n* = 3. Data were analyzed using one-way ANOVA, followed by Tukey’s *post hoc* test. Bars marked with different letters represent statistically significant differences between groups, with each letter indicating a distinct group at a significance level of *P* < 0.05. (**B**) Viral titration analysis in TCID_50_/mL for LY, VR, NADC30, SDSU73, and SD16 in supernatants of PAMs treated as described in (**A**). Values represent mean ± SD, *n* = 3. Data were analyzed using one-way ANOVA, followed by Tukey’s *post hoc* test. (**C**) Cytotoxicity analysis of different concentrations of Remdesivir incubated with PAMs for 48 h, with DMSO as the vehicle control. The red dashed line indicates 90% relative viability. The top *x*-axis illustrates DMSO concentration, expressed as the ratio of DMSO volume to medium volume (vol/vol). The bottom *x*-axis denotes compound concentrations (µM). Values represent mean ± SD, *n* = 3. (**D**) qRT-PCR analysis of SD16 RNA in PAMs treated with different concentrations of Remdesivir. DMSO was used as negative controls. Values represent mean ± SD, *n* = 3. Data were analyzed using one-way ANOVA, followed by Tukey’s *post hoc* test. (**E**) Viral titration analysis in TCID_50_/mL for SD16 in supernatants of PAMs treated as described in (D). Values represent mean ± SD, *n* = 3. Data were analyzed using one-way ANOVA, followed by Tukey’s *post hoc* test. (**F**) Representative immunofluorescence images of PAMs infected with or without PRRSV and treated with DMSO or A8-A2 at 24 h post infection. NC: non-infected cells treated with DMSO. DMSO: infected cells treated with DMSO. A8-A2: infected cells treated with 10–50 μM A8-A2. Blue represents DAPI and green represents PRRSV. Bar  =  100 µM. (**G**) The percentage of PRRSV-positive cells in images described in (F). Values represent mean ± SD, *n* = 3. Data were analyzed using one-way ANOVA, followed by Tukey’s *post hoc* test.

We next examined whether compound A8-A2 could suppress PRRSV replication in PAMs. PAMs were inoculated with type I (LY) and type II PRRSV strains [VR, NADC30, SDSU73, and high-pathogenic PRRSV SD16 (26)] for 1 h, followed by incubation with 5–50 µM of A8-A2. At 24 h post-inoculation, total RNAs were extracted from PAMs to assess PRRSV genome copy numbers, and culture media were collected for viral titration. Quantitative reverse-transcription PCR (qRT-PCR) showed that 30 or 50 µM A8-A2 significantly reduced PRRSV RNA levels across all five strains with *P* values below 0.001 (Fig. 4A). Viral titration of PAM culture supernatants further confirmed this reduction, with A8-A2 treatment at 30 or 50 µM leading to a 1.6–4.5 log drop in PRRSV titers. The effective concentration 90% (EC_90_) values of A8-A2 ranged from 10.00 to 17.92 µM for the five strains, indicating selectivity, as its CC_50_ value exceeds 50 µM with no significant cytotoxicity at this concentration (Fig. 4B). Remdesivir, a nucleotide analog with broad-spectrum antiviral activity against both epidemic and zoonotic viruses, including human coronaviruses and PRRSV (27–29), was included as a positive control. Remdesivir treatment at gradient concentrations from 1-50 µM caused a slight reduction in cell viability, to approximately 80% (Fig. 4C). In infection assays, Remdesivir significantly reduced viral RNA levels at concentrations of 1–10 µM, resulting in a 3 log reduction in viral titers, comparable to the effect of A8-A2 (Fig. 4D and E). To further validate the effect of A8-A2, we assessed PRRSV nucleocapsid (N) protein expression via immunofluorescence in PAMs infected with SD16 strain as a representative. Treatment with 10 and 20 µM A8-A2 reduced the numbers of PRRSV-positive cells compared to DMSO-treated controls, with no detectable PRRSV observed in cells treated with 30 or 50 µM A8-A2 (Fig. 4F). The percentage of PRRSV-positive cells decreased significantly in a dose-dependent manner with A8-A2 treatment (Fig. 4G). In conclusion, compound A8-A2 provided cross-protection against multiple PRRSV strains while remaining within a safe range.

### Conservation of the NendoU catalytic region across PRRSV strains

**Fig 5:**
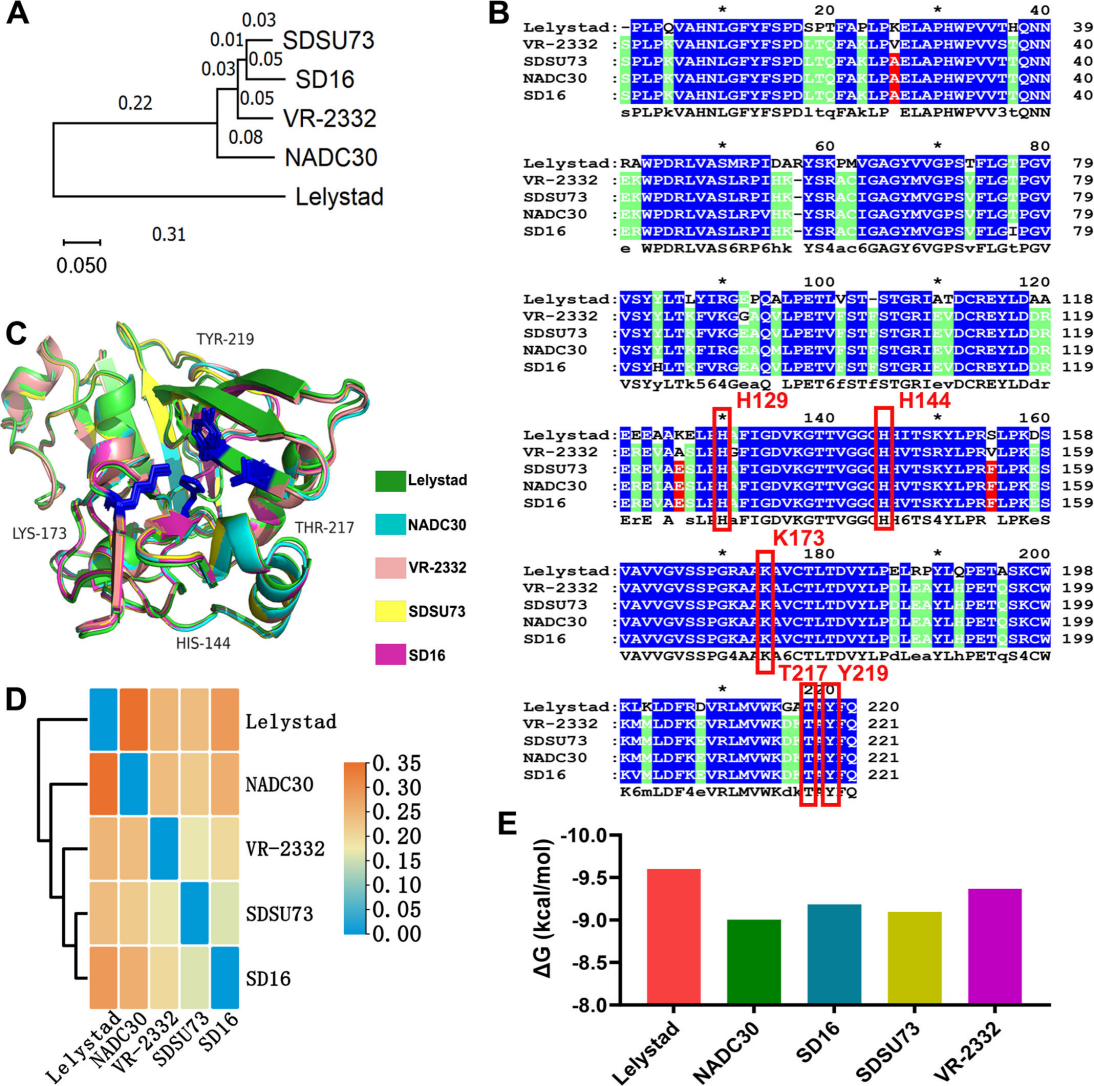
Conservation of the NendoU catalytic region across PRRSV strains. (**A**) Neighbor-joining tree representing the whole-genome phylogeny of various PRRSV strains. (**B**) Sequence alignment of the catalytic regions of NSP11 across different PRRSV strains. Blue marks indicate completely conserved sequences, green marks indicate 80% conservation, and red marks indicate 60% conservation. The key residues critical for NSP11 NendoU catalytic activity are highlighted with red frames. (**C**) Three-dimensional structural alignment of NSP11 proteins from various PRRSV strains. (**D**) Pairwise comparison of root mean squared deviation (RMSD) values based on NSP11 protein folding across PRRSV strains. (**E**) Comparison of changes in the total binding energy during molecular docking and dynamics simulations between NSP11 from various PRRSV strains and A8-A2.

To evaluate the conservation of the NendoU domain in PRRSV, we performed genetic and protein analysis across various strains. A Neighbor-Joining tree representing whole-genome phylogeny highlighted the genetic similarities among these strains (Fig. 5A). Amino acid sequence alignment of the NSP11 catalytic regions revealed full conservation of key residues essential for NendoU catalytic activity across strains, including those forming hydrogen bonds with A8-A2, such as His144, Lys173, and Thr217 (Fig. 5B). NSP11 protein folding showed highly similar structures, especially around the active sites, with low pairwise root mean squared error (RMSD) values (Fig. 5C and D). Molecular docking, followed by dynamics simulations, demonstrated that NSP11 from different PRRSV strains exhibits similar changes in total binding energy when interacting with A8-A2 (Fig. 5E). These findings demonstrate that the NendoU domain is conserved among both type I and type II PRRSV strains, particularly around catalytic sites and suggest that A8-A2 could potentially inhibit infections caused by various PRRSV strains.

### Exploring the broad-spectrum antiviral potential of A8-A2 against nidoviruses

**Fig 6:**
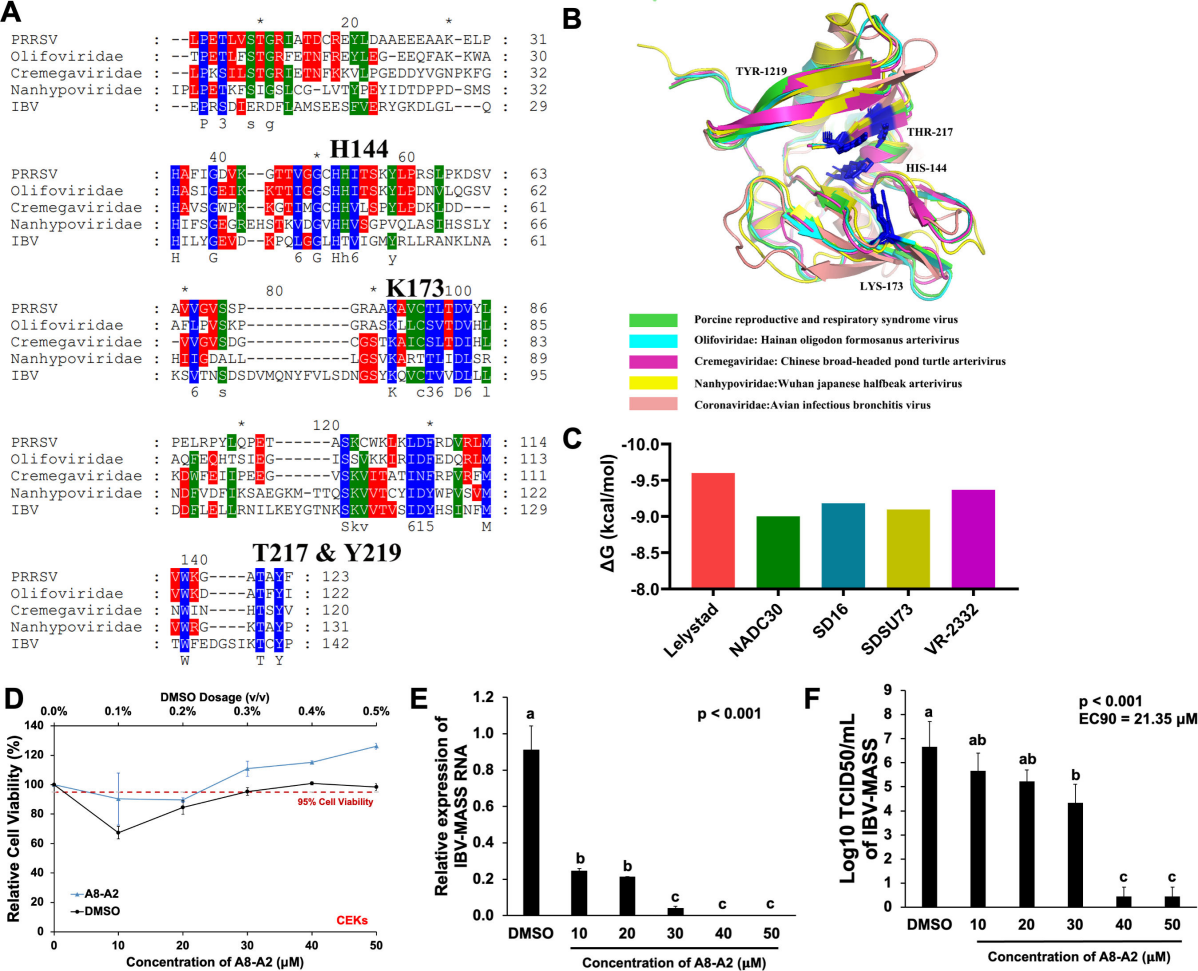
Conservation of the NendoU catalytic region across nidoviruses. (**A**) Sequence alignment of the catalytic regions of NendoU for different nidoviruses. Blue marks indicate completely conserved sequences, green marks indicate 80% conservation, and red marks indicate 60% conservation. (**B**) Three-dimensional structural alignment of NendoU-contained proteins for different nidoviuses. (**C**) Comparison of changes in the total binding energy during molecular docking and dynamics simulations between NendoU-contained proteins from different nidoviruses and A8-A2. (**D**) Cytotoxicity analysis of CEK cells treated with different concentrations of A8-A2, with DMSO as the vehicle control. The red dashed line indicates 95% relative viability. The top *x*-axis illustrates DMSO concentration, expressed as the ratio of DMSO volume to medium volume (vol/vol). The bottom *x*-axis denotes compound concentrations (µM). Values represent mean ± SD, *n* = 3. (**E**) qRT-PCR analysis of IBV-MASS RNA contents in CEK cells treated with different concentrations of A8-A2 or vehicle control DMSO. Values represent mean ± SD, *n* = 3. Data were analyzed using one-way ANOVA, followed by Tukey’s *post hoc* test. Bars marked with different letters represent statistically significant differences between groups, with each letter indicating a distinct group at a significance level of *P* < 0.05. (**F**) Viral titration analysis in TCID_50_/mL for IBV-MASS in supernatant of CEK cells treated as described in (**E**). Values represent mean ± SD, *n* = 3. Data were analyzed using one-way ANOVA, followed by Tukey’s *post hoc* test.

NendoU, a conserved endoribonuclease, is found throughout the *Nidovirales* order, which includes families such as *Arteriviridae* and *Coronaviridae*. To assess the cross-species inhibitory potential of A8-A2, we aligned amino acid sequences of NendoU catalytic regions in three arteriviruses (*Olifoviridae*: Hainan oligodon formosanus arterivirus, *Cremegaviridae*: Chinese broad-headed pond turtle arterivirus, and *Nanhypoviridae*: Wuhan Japanese halfbeak arterivirus) and one coronavirus (chicken infectious bronchitis virus, IBV). Despite low overall sequence similarity, all key residues essential for NendoU catalytic activity were highly conserved among these viruses (Fig. 6A). Structural analysis further showed notable similarities in protein folding, particularly around the catalytic sites (Fig. 6B). Molecular docking and dynamics simulations confirmed strong binding interactions between A8-A2 and NendoU across these viruses, suggesting promising inhibitory potential (Fig. 6C).

To evaluate the antiviral effects of A8-A2 beyond PRRSV, we tested it against IBV-Massachusetts (IBV-MASS) strain, known for causing severe disease and shell-less egg syndrome in chicken (30). In cytotoxicity assays, A8-A2 showed no toxic effects on chicken embryonic kidney (CEK) cells, the primary targets for IBV infection (Fig. 6D) (31). We then infected CEK cells with IBV-MASS and treated them with A8-A2 at concentrations ranging from 10 to 50 µM. qRT-PCR analysis revealed a dose-dependent reduction in IBV RNA levels (Fig. 6E). Additionally, A8-A2 treatment reduced viral titers substantially, with decreases of 2–6 logs at concentrations of 30–40 µM (Fig. 6F). These findings underscore the potential of A8-A2 as a broad-spectrum antiviral agent against nidoviruses, supporting its therapeutic application for infections beyond PRRSV.

### Impact of A8-A2 on PRRSV replication and host immune response

**Fig 7:**
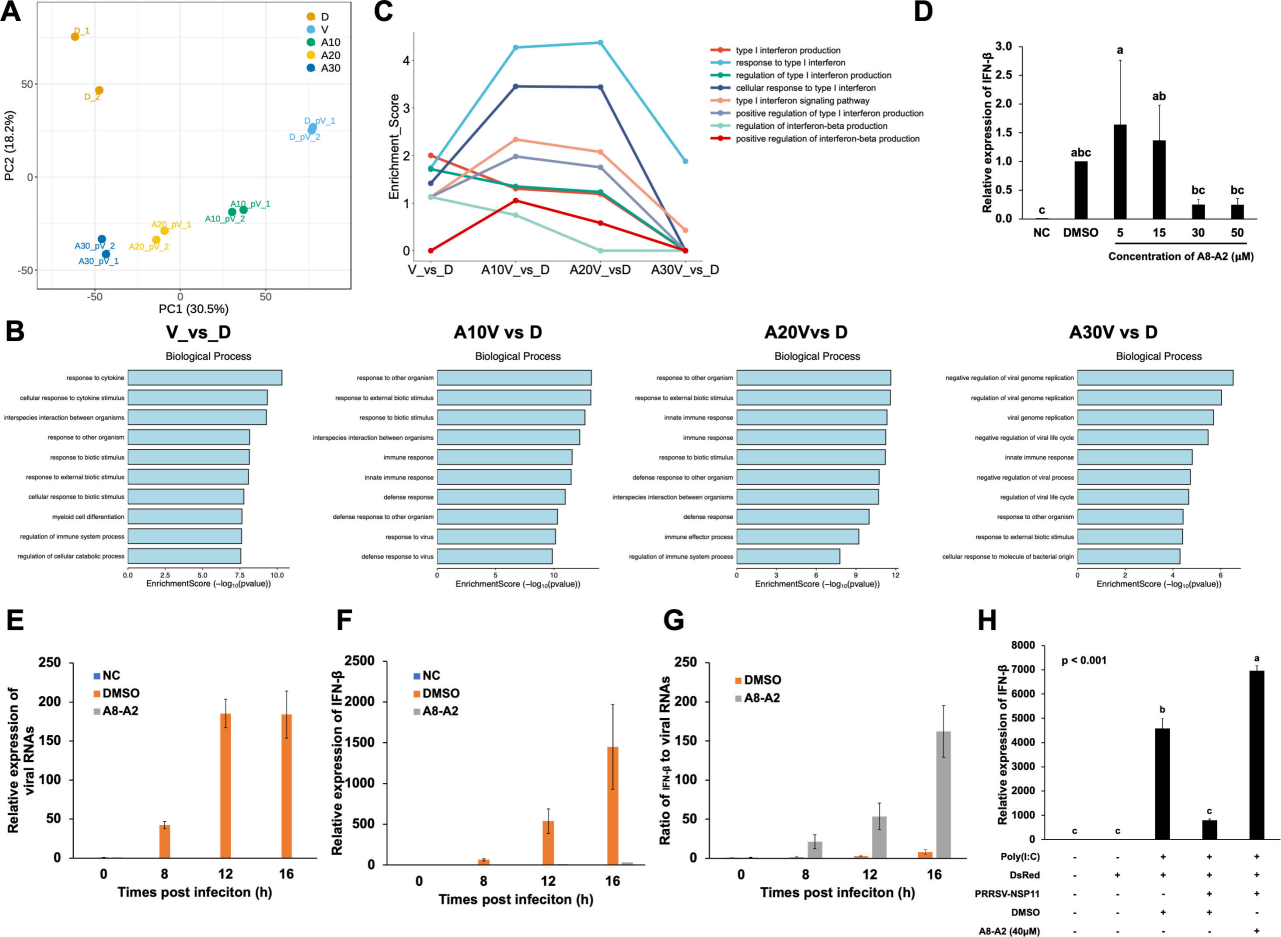
Impact of A8-A2 on PRRSV replication and host immune response. (**A**) PCA of RNA-seq data. D: non-infected cells treated with DMSO. V: PRRSV SD16 infected cells treated with DMSO. A10V, A20V, and A30V: PRRSV SD16-infected cells treated with 10, 20, or 30 µM A8-A2. (**B**) Biological process analysis of RNA-seq data for the groups described in (**A**). (**C**) Differential and GO enrichment analysis of RNA-seq data for the groups described in (**A**). (**D**) qRT-PCR analysis of IFN-β expression in PAMs infected with or without PRRSV and treated with DMSO or A8-A2 at 24 h after infection. NC: non-infected cells treated with DMSO. DMSO: infected cells treated with DMSO. A8-A2: infected cells treated with 5–50 μM A8-A2. Values represent mean ± SD, *n* = 3. Data were analyzed using one-way ANOVA, followed by Tukey’s *post hoc* test. Bars marked with different letters represent statistically significant differences between groups, with each letter indicating a distinct group at a significance level of *P* < 0.05. (**E**) qRT-PCR analysis of viral RNA contents in PAMs infected with or without PRRSV and treated with or without A8-A2 at 0, 8, 12, and 16 h after infection. NC: non-infected cells treated with DMSO. DMSO: infected cells treated with DMSO. A8-A2: infected cells treated with 40 µM A8-A2. Values represent mean ± SD, *n* = 3. (**F**) qRT-PCR analysis of IFN-β expression in PAMs infected with or without PRRSV and treated with or without A8-A2 at 0, 8, 12, and 16 h after infection. NC: non-infected cells treated with DMSO. DMSO: infected cells treated with DMSO. A8-A2: infected cells treated with 40 µM A8-A2. Values represent mean ± SD, *n* = 3. (**G**) Ratio of viral RNA contents from (**E**) to IFN-β expression from (**F**) in PAMs infected with or without PRRSV and treated with or without A8-A2 at 0, 8, 12, and 16 h after infection. NC: non-infected cells treated with DMSO. DMSO: infected cells treated with DMSO. A8-A2: infected cells treated with 40 µM A8-A2. Values represent mean ± SD, *n* = 3. (**H**) qRT-PCR analysis of IFN-β in HEK293T cells expressing either DsRed or DsRed-PRRSV-NSP11, transfected with PBS control or Poly(I:C) and treated with DMSO control or 40 µM A8-A2. “+” represents presence and “−” indicates absence of proteins or chemicals. Values represent mean ± SD, *n* = 3. Data were analyzed using one-way ANOVA, followed by Tukey’s *post hoc* test.

To investigate the effect of A8-A2 impact on PRRSV-infected cells, we conducted total RNA sequencing (RNA-seq) on PAMs infected with PRRSV and treated with different concentrations of A8-A2. Principal component analysis (PCA) of the RNA-seq data revealed a clear separation along the PC1 axis between control and PRRSV-infected groups. Notably, PAMs treated with 10, 20, and 30 µM A8-A2 showed a concentration-dependent shift toward the control group, suggesting that A8-A2 progressively altered the transcriptome profile from a viral infection status to one indicative of viral clearance (Fig. 7A). Gene ontology (GO) analysis revealed that while the PRRSV-infected group exhibited strong enrichment in immune response pathways, the cells treated with 30 µM A8-A2 showed significant enrichment in pathways related to the viral suppression, indicating effective antiviral activity (Fig. 7B). Further differential and GO enrichment analysis in type I IFN pathways revealed that in PRRSV-infected cells, the host response to type I IFN was elevated at 10 and 20 µM A8-A2 but returned to near-control levels at 30 µM (Fig. 7C). qRT-PCR analysis on total RNAs from PAMs infected with various PRRSV strains comfirmed a positive correlation between IFN-β levels and the reduction in viral titers following A8-A2 treatment (Fig. 7D; Fig. S3A through C).

To explore whether the inhibition of NendoU activity by A8-A2 directly affects PRRSV replication, we monitored PRRSV replication over time in infected PAMs. In the control group treated with DMSO, PRRSV replication increased significantly from 8 to 16 h post-inoculation (Fig. 7E), accompanied by a gradual rise in the cellular IFN-I response (Fig. 7F). In contrast, A8-A2-treated cells showed no viral replication after inoculation (Fig. 7E), and virtually no IFN-β response was detected from 0 to 16 h (Fig. 7F). These results indicate that A8-A2 effectively blocks PRRSV replication in host cells, consistent with the known role of NSP11 in PRRSV replication.

PRRSV NendoU not only promotes viral replication, but also inhibits cellular sensing of viral dsRNA, thereby suppressing IFN-I production to evade host immune defenses (32, 33). We hypothesized that by inhibiting NendoU, A8-A2 could restore the cellular IFN-I response. When normalized by viral RNA levels, IFN-β expression increased with A8-A2 treatment (Fig. 7G). Given that Poly(I:C), a double-stranded RNA molecule, activates IFN-I pathway and NSP11 endoruibonuclease activity suppresses Poly(I:C)-induced IFN-β promoter activity (18), we evaluated the effects of A8-A2 on PRRSV-NSP11 in Poly(I:C)-induced IFN-β expression in 293T cells. This approach allowed us to specifically assess the effect of A8-A2 without interference from viral content. While Poly(I:C) stimulation induced strong IFN-β expression in control cells, this response was significantly suppressed by PRRSV-NSP11 overexpression. Remarkably, treatment with A8-A2 completely restored IFN-β expression in these cells (Fig. 7H), while cells treated with A8-A2 alone showed negligible IFN-β levels (Fig. S3D). Taken together, these results suggest that A8-A2 not only inhibits direct viral replication but also reactivates the cellular IFN-I response through the inhibition of NendoU activity.

### Evaluating spliceosome inhibition in the antiviral mechanism of A8-A2

**Fig 8:**
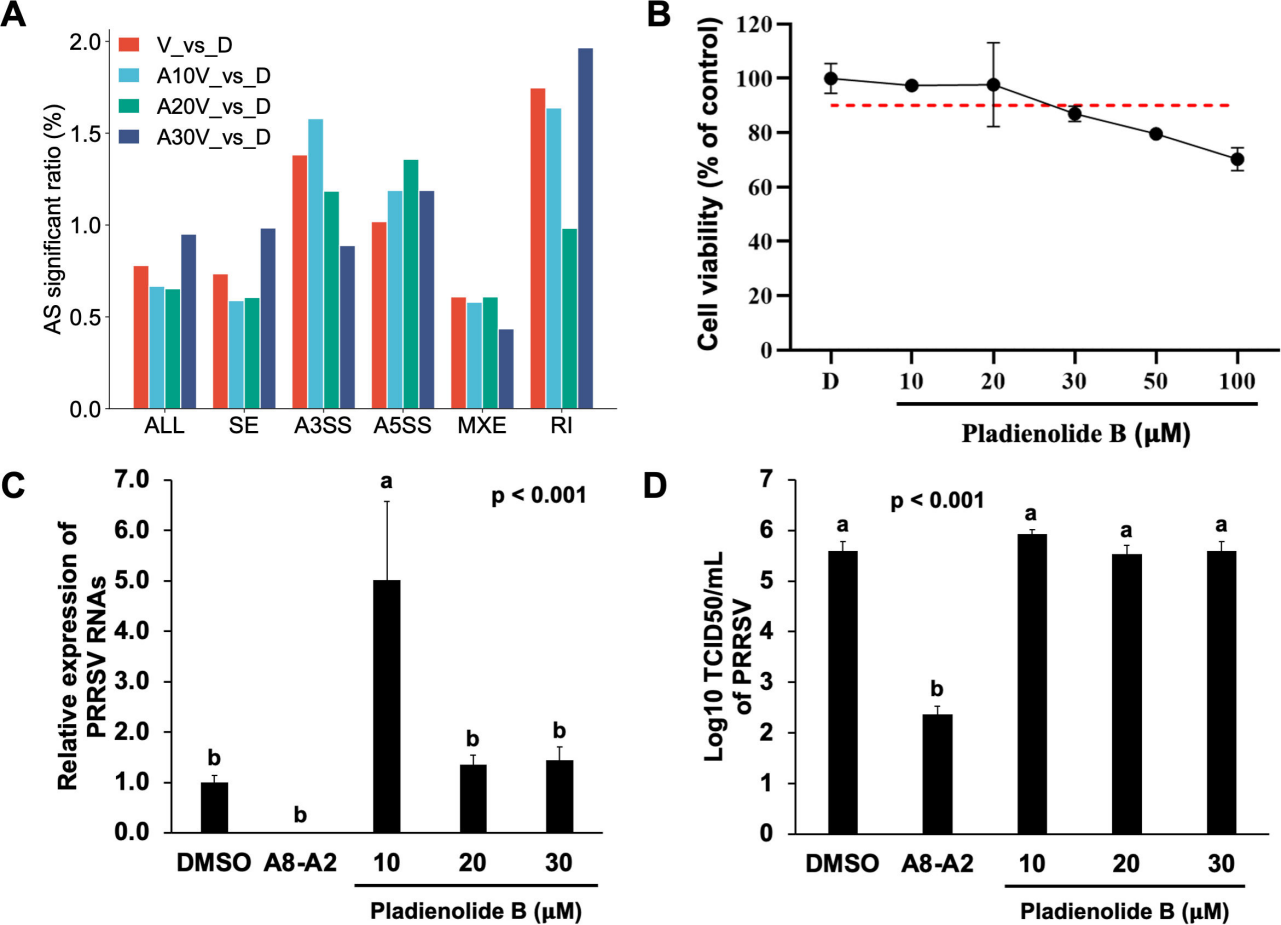
The effect of A8-A2 on RNA spliceosome activity. (**A**) Analysis of alternative splicing events including SE, MXE, A5SS, A3SS, and RI from RNA-seq data. D: non-infected cells treated with DMSO. V: PRRSV SD16-infected cells treated with DMSO. A10V, A20V, and A30V: PRRSV SD16-infected cells treated with 10, 20, or 30 µM A8-A2. (**B**) Cytotoxicity analysis of Pladienolide B at various concentrations in PAMs at 48 h, with DMSO as the vehicle control. The red dashed line indicates 90% relative cell viability. Values represent mean ± SD, *n* = 3. (**C**) qRT-PCR analysis of SD16 RNA in PAMs treated with different concentrations of Pladienolide B, with DMSO as the negative control and A8-A2 as the positive control. Values represent mean ± SD, *n* = 3. Data were analyzed using one-way ANOVA, followed by Tukey’s *post hoc* test. Bars marked with different letters represent statistically significant differences between groups, with each letter indicating a distinct group at a significance level of *P* < 0.05. (**D**) Viral titration analysis (TCID_50_/mL) of SD16 in the supernatant of PAMs treated as described in (**C**). Values represent mean ± SD, *n* = 3. Data were analyzed using one-way ANOVA, followed by Tukey’s *post hoc* test.

Given that A8-A2 is an analog of Madrasin, a previously reported spliceosome inhibitor (24), we hypothesized that its antiviral effects mignt stem from interference mRNA splicing. To explore this possibility, we analyzed various alternative splicing events—including Skipped Exon (SE), Mutually Exclusive Exon (MXE), Alternative 5′ Splice Site (A5SS), Alternative 3′ Splice Site (A3SS), and Retained Intron (RI)—in PRRSV-infected cells treated with different concentrations of A8-A2. In all samples, including those treated with A8-A2, the frequency of alternative splicing events remained stable, with only about a 1% variation compared to the DMSO-treated group. This stability suggests that A8-A2 does not significantly alter alternative splicing in a way that would impact viral infection (Fig. 8A). To further test this, we examined the effects of Pladienolide B (34), a well-established spliceosome inhibitor, on PRRSV infection. While Pladienolide B reduced cell viability in a dose-dependent manner at concentrations from 10 to 100 µM (Fig. 8B), it did not inhibit PRRSV replication. Interestingly, viral RNA levels even increased at the 10 µM concentration (Fig. 8C and D). These findings suggest that a host cell spliceosome inhibition does not suppress PRRSV replication, indicating the antiviral activity of A8-A2 is independent of mRNA splicing inhibition.

## DISCUSSION

PRRSV virus poses a significant challenge to swine health and the pig industry worldwide. Despite extensive efforts to develop prophylactic and therapeutic measures over the past three decades, effective treatment for PRRSV remains elusive. This difficulty is primarily due to the high genetic, pathogenic, and antigenic variability among PRRSV strains (35–37). Consequently, there is an urgent need for effective strategies to control this virus. Here, we report for the first time the identification of small molecules that effectively inhibit PRRSV infections by targeting the NendoU enzymatic function of the NSP11 protein, which is crucial for the viral replication in host cells.

Our findings demonstrate that A8-A2 significantly inhibits the NendoU activity of PRRSV NSP11 and binds directly to this protein. A8-A2 showed a reduced affinity for NSP11 mutants at enzyme activity sites, supporting its specific targeting of the NSP11 functional domain. A8-A2 displayed a dose-dependent inhibitory effect on PRRSV infection in PAMs and was effective against various PRRSV strains, including both type I and type II. This broad activity is particularly valuable, given the genetic diversity and rapid evolution characteristic of PRRSV. Additionally, A8-A2 displayed antiviral activity over another nidovirus IBV which exhibits conservation of catalytic sites of NendoU. Although further testing across additional viruses is intriguing, these findings highlight the potential of A8-A2 as a broad-spectrum antiviral agent against nidoviruses.

Importantly, our data indicate that A8-A2 inhibits viral replication at early stages, with minimal antiviral signaling detected in host cells at high concentrations, underscoring its potential as a robust antiviral compound. Moreover, A8-A2 was also shown to reverse the Poly(I:C)-induced IFN inhibition caused by NSP11 overexpression, confirming its direct effect on NSP11. Although A8-A2 and related compounds share structural similarities with Madrasin, a pre-mRNA splicing inhibitor reported previously (24), a comprehensive literature review found no prior reports of antiviral activity for A8-A2 and few follow-up studies with even contrasting results on the splicing inhibition on Madrasin (38). Since PRRSV does not rely on RNA splicing for its viral protein expression and another well-studied splicing inhibitor—Pladienolide B showed no anti-PRRSV effect, A8-A2 is unlikely to exert its antiviral effects through spliceosome inhibition.

While our findings are promising, several limitations require further investigation. First, this study primarily relied on *in vitro* assays; therefore, the efficacy and safety of A8-A2 *in vivo* remain to be determined. Further research should assess whether A8-A2 can inhibit the replication of other nidoviruses beyond IBV, potentially broadening its application as a general anti-nidoviral agent. Moreover, the specificity of A8-A2 for PRRSV NSP11 requires a thorough evaluation, as off-target effects on host enzymes or other viral proteins could lead to unintended effects. Finally, conducting a structure-activity relationship analysis could optimize the potency and selectivity of A8-A2, enhancing its potential as a viable antiviral therapeutic.

In conclusion, our study identifies A8-A2 as a potent NendoU inhibitor with broad-spectrum antiviral efficacy. These findings elucidate a novel therapeutic approach for combating PRRSV and other nidiviruses.

## MATERIALS AND METHODS

### Chemicals, cells, viruses, and plasmids construction

All screening compounds were purchased from ChemBridge Corporation (CA, USA). PAMs were harvested from healthy, PRRSV-negative Landrace/Yorkshire cross pigs aged 4–6 months. Briefly, the pigs were slaughtered, and their lungs were transferred on ice to a cell culture cabinet. Warm phosphate-buffered saline (PBS) containing 200 U/mL penicillin and 200 µg/mL streptomycin was carefully injected through the trachea into the bronchi on both sides of the lungs. The lungs were then massaged, and bronchoalveolar lavage fluid (BALF) was retrieved. The BALF was centrifuged at 400 × *g* for 15 min, and the pellets were washed two times with warm complete medium. Cells were counted and frozen in 90% fetal bovine serum (FBS) and 10% DMSO and stored in liquid nitrogen. PAMs were cultivated in RPMI-1640 supplemented with 10% FBS, 2 mM GlutaMAX Supplement, 0.1 mM MEM non-essential amino acids, 1 mM sodium pyruvate, 100 U/mL penicillin, 100 µg/mL streptomycin, and 0.5 µg/mL Amphotericin B. Pig CD163 (pCD163) was amplified from the pig genome and inserted into the pMXs retroviral vector (Cell Biolabs, CA, USA). To establish a pCD163-expressing Marc-145 cell line (pCD163-Marc-145), a pCD163-expressing retrovirus was produced. Briefly, pMXs-pCD163 was co-transfected with pUMVC and pCMV-VSV-G (Addgene, MA, USA) packaging plasmids into HEK293T cells using Fugene 6 (Promega, WI, USA). Supernatants containing the virus were collected at 48 and 72 h post-transfection, following previously described protocols (39). Marc-145 cells were cultivated in FP medium (DMEM containing 10% FBS, 2 mM GlutaMAX Supplement, 0.1 mM MEM non-essential amino acids, 50 U/mL penicillin, and 50 µg/mL streptomycin). These cells were then incubated with the pCD163-expressing retrovirus at 32°C while being centrifuged at 650 × *g* for 45 min. The infection process was repeated after 24 h. Following the second infection, the cells were stained with CD163 Monoclonal Antibody (2A10/11), PE (1:10, ThermoFisher Scientific, MA, USA) at 24 h post-infection. Fluorescence-positive cells were sorted using a FACSAria II Cell Sorter within a BSL2 Enclosure (BD, NJ, USA). All PRRSV strains were propagated and titrated in pCD163-MARC-145 cells, following previously described protocols (40, 41).

To establish DsRed and DsRed-NSP11-expressing cell lines (HEK293T-DsRed and HEK293T-DsRed-NSP11), DsRed was amplified from the pMXs-DsRed plasmid, while PRRSV NSP11 was amplified from the viral genome of PRRSV LY. Both were inserted into the Tet-O-FUW vector. For lentivirus packaging, HEK293T cells were co-transfected with the packaging plasmids psPAX2 and pCMV-VSV-G (Addgene, MA, USA), along with either FUW-M2rtTA (Addgene, MA, USA), Tet-O-FUW-DsRed, or Tet-O-FUW-DsRed-NSP11, using Fugene 6 (Promega, WI, USA). The virus-containing supernatants were collected at 48 and 72 h post-transfection. HEK293T cells were cultured in six-well plates and co-incubated with FUW-M2rtTA and lentiviruses expressing DsRed or DsRed-NSP11 in the presence of 10 µg/mL polybrene for 24 h.

For protein expression, the NSP11 from VR-2332 and Lelystad (VR-NSP11 and LY-NSP11) were amplified from reverse-transcribed viral genome and cloned into pET6xHN-N (Takara, CA, USA) vector. Mutant VR-NSP11 at H144A/T217A(VR-mNSP11) and mutant LY-NSP11at H145A/T218A(LY-mNSP11) were cloned into pET6xHN-N Vector by multiple PCR fragments cloning with In-Fusion cloning kit (Takara, CA, USA). All cloning primers are listed in Table S2.

### Protein expression and purification

The recombinant pET6xHN-N constructs were transformed into *Escherichia coli* strain BL21 (DE3) (New England Biolabs, MA, USA). The transformed clones were cultured at 37°C in Luria-Bertani (LB) broth containing 100 mg/mL carbenicillin. Protein expression was induced by adding 1 mM isopropyl β-d-1-thiogalactopyranoside (IPTG) when the optical density at 600 nm (OD_600_) reached 0.8–1.0, followed by incubation at 37°C for 1–3 h. For protein purification, the cells were harvested by centrifugation at 5,000 rpm for 15 min and resuspended in xTractor Buffer containing DNase I, lysozyme solution, and a protease inhibitor cocktail (Takara Bio USA, Inc., CA, USA). The cell suspension was sonicated three times for 10 s each, with 30 s pauses in between, and then centrifuged at 10,000 rpm for 20 min. The supernatant was incubated with equilibrated TALON Metal Affinity Resin (Takara Bio USA, Inc., CA, USA) for 20 min on ice. Proteins were eluted from the resin using an elution buffer (pH 7.0, 150 mM imidazole, 50 mM NaH2PO4, and 300 mM NaCl). The eluted proteins were then concentrated and buffer-exchanged to PBS (pH 7.4) using a protein concentrator PES, 10K MWCO (Pierce Biotechnology, PA, USA).

### *In-silico* screen of the ChemBridge small chemical library

The crystal structure of PRRSV NSP11 (14, 15) was used for our in-silico docking-based virtual screening using Glide from Schrodinger-2024–2 (42). The catalytic site of the PRRSV NSP11 enzyme protein was defined using the default parameters for receptor-grid generation in Schrodinger-2024-2 (43). Once the receptor grid was generated, a stepwise high-throughput virtual screen was performed with ~155K structures from our in-house chemical library (ChemBridge). All compound structural coordinates were retrieved in the sdf format. Structural coordinates were converted into the three-dimensional (3D) format and were energetically minimized using the OPLS4 force field at pH 7.4 (44). The chiral centers of all the ligand molecules were selected to retain the original state, in order to avoid stereoisomer generation. Compounds structures were modified to ensure that their protonation state was the same as it would be at physiological pH (7.4). Iterative docking-based virtual screening was carried out using Schrodinger suite Glide module. The *in-silico* protocol is comprised three steps: high-throughput virtual screening, standard precision (SP), and extra precision (XP). Compounds were assigned a Glide score (45) that predicts their ability to bind the NSP11 catalytic site and structures that meet a pre-determined threshold were advanced to the next round of screening. Compounds that exhibited a Glide Score of −7.0 in the most strenuous XP screening were chosen for experimental analysis.

### NendoU enzyme activity assay

The NendoU enzyme activity assay was designed based on the fluorescence resonance energy transfer technique. Specifically, a 23-mer RNA oligonucleotide named R23.1 (5′-6-[FAM]-UCUAAACGAAGCGAAACCCUAAG-[BHQ1]-3′) was synthesized by Sigma-Aldrich, Inc. VR-NSP11 or LY-NSP11 proteins were incubated with HKD buffer (containing 50 mM HEPES [pH 7.5], 50 mM KCl, and 1 mM dithiothreitol in 0.1% DEPC-treated water), 1 µM R23.1, and H_2_O to a total volume of 50 µL for testing enzyme activity. Additionally, 15 μM of a compound was added for inhibition screening. Fluorescence intensity was measured at an excitation wavelength of 492 nm and an emission wavelength of 520 nm every 15 min for 120 min at 25°C. For optimization of NendoU activity, 0.3–2.4 μM VR-NSP11 and 0.13–1.04 μM LY-NSP11 were tested. The final chemical screening assays utilized 1.2 µM VR-NSP11 and 0.52 µM LY-NSP11. The IC_50_ was calculated from fluorescence intensity following treatment with different concentrations of A8-A2 at 2 h using the equation: [Inhibitor] versus response in GraphPad Prism.

### Cytotoxicity assay

The cytotoxicity of the selected screening compounds was evaluated in PAMs from different donors using the MTT assay kit, following the manufacturer’s instructions (In Vitro Toxicology Assay Kit, MTT-based, Sigma-Aldrich). Briefly, the cells were cultured in 48-well plates and incubated with 5–50 μM of the compounds, diluted from a 10 mM stock solution dissolved in 100% DMSO, or with corresponding doses of DMSO as the vehicle control, for 48 h at 37°C. Following incubation, 20 µL of the MTT assay labeling reagent was added to each well and incubated for 4 h. Subsequently, 200 µL of the solubilization solution was added to each well to fully dissolve the formazan crystals through overnight incubation. The absorbance of the samples was measured at 550 nm using the CLARIOstar Plus plate reader (BMG LABTECH, NC, USA). The absorbance of untreated cells was used as a reference to normalize relative cell viability.

### Quantitative reverse transcription-PCR

Total RNA was extracted from the cells using the RNeasy Mini Kit (Qiagen, MD, USA) following the manufacturer’s instructions. RNA concentrations were measured using a Nanodrop spectrophotometer (Thermo Fisher Scientific). Equal amounts of RNA were then reverse transcribed into cDNA using the iScript cDNA Synthesis Kit (Bio-Rad, CA, USA). Specific qRT-PCR primers for the five PRRSV strains, porcine GAPDH (pGAPDH), porcine IFN-β (pIFN-β), IBV and chicken GAPDH (ckGAPDH) were designed based on genome sequences published in NCBI, to detect genomic RNA (virus) or mRNA (cells), as listed in Table S3. GAPDH served as the housekeeping gene, and the DMSO-treated group was used as the reference sample for gene expression normalization.

### PRRSV and IBV infection and titration assay

For the PRRSV infection assay, PAMs were seeded in 24-well plates and inoculated with LY, VR, NADC30, SDSU73, or SD16 strains (multiplicity of infection [MOI] = 0.05) for 1 h. After removing the virus inoculum, the cells were treated with A8-A2, Remdesivir, Pladienolide B, or DMSO as a vehicle control. Three biological replicates were conducted using PAMs from different donors. For IBV infection assay, CEK cells were cultured in 24-well plate and infected with IBV-MASS strain for 1 h, followed by treatment of A8-A2 or DMSO as a vehicle control. In titration assay, pCD163-Marc-145 or NCI-H1299 was seeded in 48-well plates and grown to approximately 80% confluency. The collected cell culture media were prepared by 10-fold serial dilution, and 100 µL of each dilution was added per well in six replicates. After a 2-h incubation, the inoculum was removed and replaced with 0.5 mL of medium containing 2% FBS. The cells were cultured at 37°C for 4–6 days for observation of cytopathic effects. The viral titer was calculated using the Reed and Müench method and expressed as the median tissue culture infectious dose per milliliter (TCID_50_/mL). The EC_90_ value, the inhibitor concentration required to reduce the viral titer by 90% (1 log10), was determined using the equation [Inhibitor] versus response in GraphPad Prism.

### MST assay

The VR/LY-NSP11 and VR/LY-mNSP11 proteins were labeled using either the Monolith Protein Labeling Kit RED-NHS Second Generation or the Monolith His-Tag Labeling Kit RED-tris-NTA 2nd Generation (NanoTemper Technologies, Inc., CA, USA) according to the manufacturer’s instructions. The labeled proteins were then mixed with serially diluted compounds. These mixtures were loaded into capillaries, and the affinity was measured using the Monolith NT.115 (NanoTemper Technologies, Inc., CA, USA) at the specified MST power.

### Genome and protein structure alignment and molecular interaction analysis

Protein 3D structures were generated using ColabFold v1.5.5 (46). RNA sequences and NSP11 protein sequences from various virus strains were downloaded from GenBank and subsequently folded. The Rank1 structure was selected as the primary 3D representation of the protein. Molecular docking was conducted in XP mode using the Glide module in Schrödinger Suite 2024-2 (43). Molecular dynamics simulations were carried out with the Desmond module in Schrödinger Suite 2024-2 (parameters: solvent model = SPC, salt solution = 0.15 M NaCl, simulation time = 50 ns, and ensemble class = NPT). Data analysis was performed using the Simulation Interaction Diagram module. Molecular alignment and RMSD calculations were executed using the QuickAlign module in Schrödinger Suite 2024-2. PyMOL v2.5.4 was employed for visualization, and RMSD data were used to create a heat map in TBtools v2.102. Alignment and Neighbor-Joining tree construction were performed using the ClustalW module in MEGA11 (47).

### Immunofluorescence

PAMs were seeded in 12-well plates and inoculated with SD16 strains at MOI = 0.1 for 1 h. Following the removal of the virus inoculum, cells were treated with 5–50 µM A8-A2 or DMSO as a vehicle control for 24 h. The cells were then fixed with 4% paraformaldehyde for 15 min, permeabilized with 0.5% Triton X-100 for 15 min, and blocked with 5% bovine serum albumin in goat serum for 1 h at room temperature (RT). Subsequently, the cells were incubated with rabbit polyclonal antibodies against the PRRSV N protein (1:300, GeneTex, 16646-1-AP, CA, USA) for 2 h at RT. After washing with PBS, the cells were incubated with secondary antibodies conjugated to Fluorescein (FITC) (1:500, Proteintech, SA00003-2, Wuhan, China) for 1 h at RT. Finally, nuclei were counterstained using 4,6-diamidino-2-phenylindole (DAPI) (Sigma-Aldrich, MO, USA). Images of the cells were captured using the EVOS M7000 (Thermo Fisher Scientific). The percentage of PRRSV-positive cells was calculated by dividing the number of green fluorescence-positive cells (PRRSV) by the number of blue fluorescence-positive cells (DAPI).

### RNA sequencing and analysis

PAM cells were seeded in a 12-well plate and divided into the following groups: D (DMSO), V (DMSO + SD16), A10V (A8-A2 10 µM + SD16), A20V (A8-A2 20 µM + SD16), and A30V (A8-A2 30 µM + SD16). After 24 h, the cells were trypsinized, centrifuged, and washed two times with PBS. TRIzol was added, and the samples were scraped into centrifuge tubes, total RNA was extracted. The quality of the samples was assessed using the Agilent 2100 Bioanalyzer, and transcriptome sequencing was performed on the Illumina sequencing platform in Novogene Co., Ltd (Tianjin, China). The sequencing data were processed using a pipeline that included fastp, HISAT2, StringTie, and featureCounts to generate the expression matrix (RefSeeq assembly accession: GCF_000003025.6). After normalization, differential expression analysis was conducted using the DESeq2 (V1.20.0). The *P* values were adjusted using the Benjamini and Hochberg method, and adjusted *P* values ≤ 0.05 and |log2 (fold change)| ≥1 were set as the thresholds for significant differential expression. GO and KEGG enrichment analyses of differentially expressed genes were performed using the clusterProfiler software (V3.8.1). Alternative splicing event analysis was conducted using the rMATS (V4.1.0).

### IFN-β stimulation assay

HEK293T-DsRed and HEK293T-DsRed-NSP11 cells were cultured in 12-well plates and treated with 1 µg/mL doxycycline (Dox) for 24 h. Following this, cells were treated with either DMSO as a control or compound A8-A2. Subsequently, cells were transfected with 2 µg Poly(I:C) (InvivoGen, CA, USA) using Lipofectamine 3000 transfection reagent (Thermo Fisher Scientific, MA, USA). After an additional 24 h, total RNA was extracted from the cells and subjected to qRT-PCR to measure the relative expression levels of IFN-β.

### Statistical analysis

All experiments were performed independently at least three times, each with one technical replicate. The mean of three biological replicates was used for plotting. Data were analyzed using one-way ANOVA, followed by Tukey’s *post hoc* test. Results are expressed as mean ± standard deviation (SD), with a *P* value < 0.05 considered statistically significant.

## Data Availability

RNA-seq data are available at Bioproject under accession number PRJNA1185196.

## SUPPLEMENTAL MATERIAL

Supplemental figures jvi.02034-24-s0001.docx Figures S1 to S3.

Tables S2 and S3 jvi.02034-24-s0002.docx Table S2, primers for cloning; Table S3, primers for qRT-PCR.

Table S1 jvi.02034-24-s0003.xlsx In silico screening identified NSP11 interacting chemicals and A8 and C5 analogs.

ASM does not own the copyrights to Supplemental Material that may be linked to, or accessed through, an article. The authors have granted ASM a non-exclusive, world-wide license to publish the Supplemental Material files. Please contact the corresponding author directly for reuse.
