# Supplementary material for: Discovery of small molecules against porcine reproductive and respiratory syndrome virus replication by targeting NendoU activity
Source: J Virol. 2024 Dec 31;99(2):e02034-24. doi: 10.1128/jvi.02034-24 (PMC11852993; doi:10.1128/jvi.02034-24)
Supplement: Supplemental figures — Figures S1 to S3. [file jvi.02034-24-s0001.docx]

**Supplementary Figures**

**
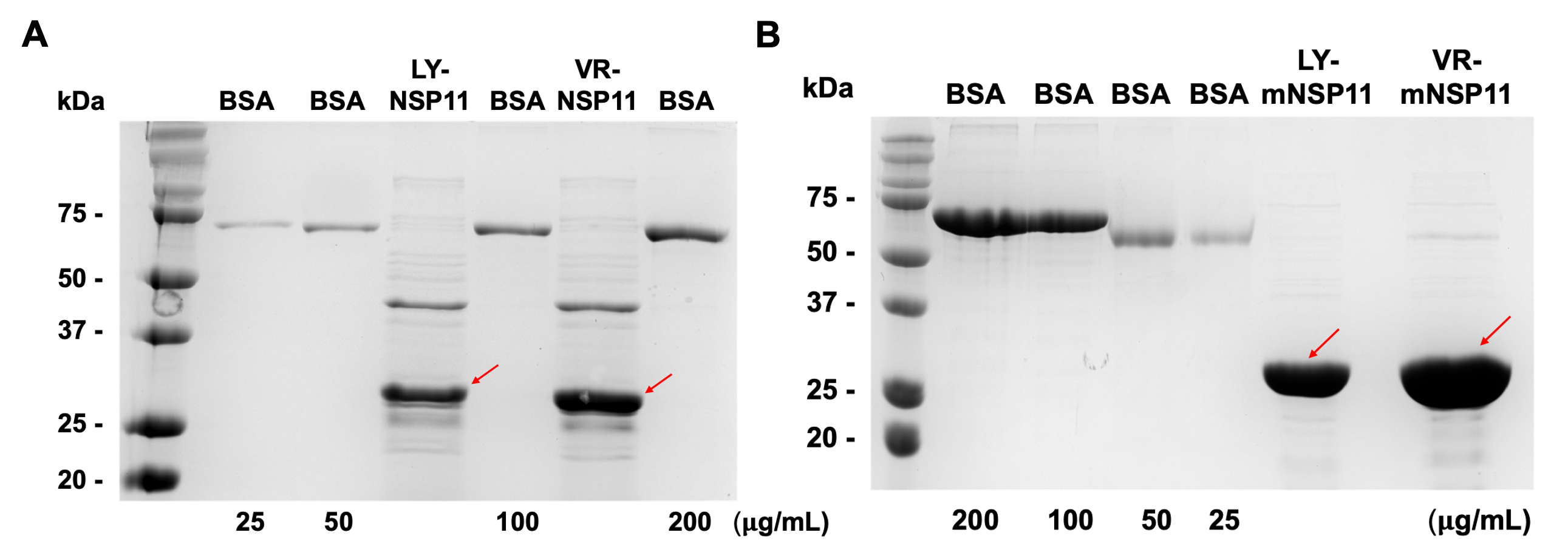
**

**Fig. S1: Purification of PRRSV NSP11 and mutated NSP11.**

1. Coomassie staining of purified recombinant proteins VR-NSP11 and LY-NSP11. Target proteins are marked with red arrows. BSA was used as the loading control.
2. Coomassie staining of purified recombinant proteins VR-mNSP11 and LY-mNSP11. Target proteins are marked with red arrows. BSA was used as the loading control.


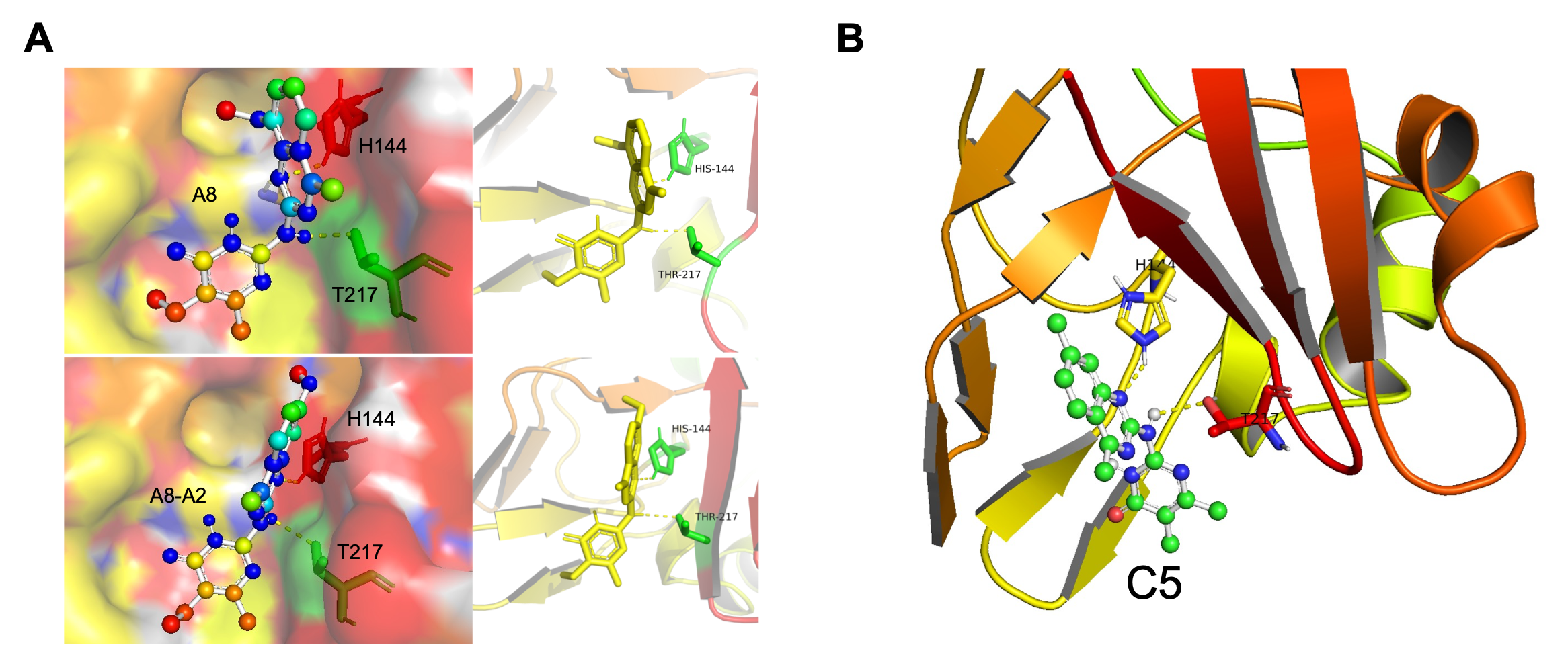


**Fig. S2: Molecular docking analysis of NSP11 with hit compounds.**

1. Molecular docking analysis depicting interactions between compounds A8 and A8-A2 with PRRSV NSP11 enzyme catalytic region.
2. Molecular docking analysis depicting potential interaction between compound C5 with the PRRSV NSP11 enzyme catalytic region.


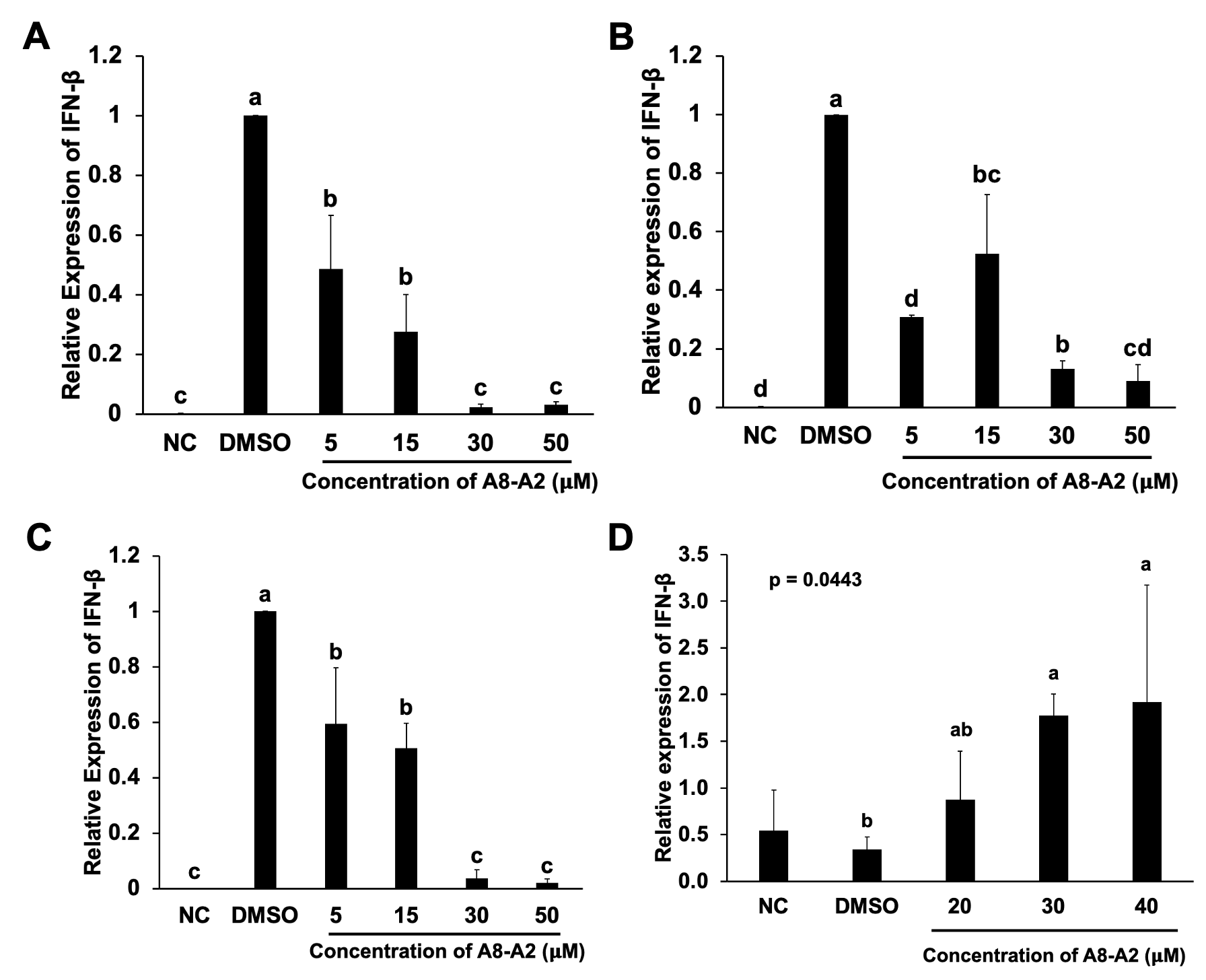


**Fig. S3: IFN-β analysis in PRRSV infected PAMs and 293T cells.**

1. qRT-PCR analysis of IFN-β expression in PAMs infected with or without PRRSV LY (A), NADC30 (B) and SDU73 (C) and treated with DMSO or A8-A2 at 24 h after infection. NC: non-infected cells treated with DMSO. DMSO: infected cells treated with DMSO. A8-A2: infected cells treated with 5-50 μM A8-A2. Values represent Mean±SD, n=3. Data were analyzed using one-way ANOVA, followed by Tukey's post-hoc test. Bars marked with different letters represent statistically significant differences between groups, with each letter indicating a distinct group at a significance level of p < 0.05.
2. See A)
3. See A)
4. qRT-PCR analysis of IFN-β expression in 293T cells treated with DMSO or A8-A2 alone. NC: non-infected cells treated with DMSO. DMSO: infected cells treated with DMSO. 20-40 μM A8-A2: infected cells treated with A8-A2. Values represent Mean±SD, n=3. Data were analyzed using one-way ANOVA, followed by Tukey's post-hoc test.
