# Supplementary material for: Discovery of small molecules against porcine reproductive and respiratory syndrome virus replication by targeting NendoU activity
Source: J Virol. 2024 Dec 31;99(2):e02034-24. doi: 10.1128/jvi.02034-24 (PMC11852993; doi:10.1128/jvi.02034-24)
Supplement: Tables S2 and S3 — Table S2, primers for cloning; Table S3, primers for qRT-PCR. [file jvi.02034-24-s0002.docx]

**Table S2. Primers for Cloning**

| **Cloning Primers Table** | | | |
| --- | --- | --- | --- |
| **Construct** | **Gene** |  | **Sequence (5’ → 3’)** |
| PMXs-pCD163 | pCD163 | Forward | **AGTTAATTAAGGATCC**  ATGGTGCTACTTGAAGACTCTGGATC |
|  |  | Reverse | **ACTGTGCTGGCGGCCGC**  TCATTGTACTTCAGAGTGGTCTCCTGA |
| pET6xHN-N-VR-NSP11 | VR-NSP11 | Forward | **GATAAGGCCTCTGTCGAC** GGGTCGAGCTCTCCGCTC |
|  |  | Reverse | **AAGCGGCCGCCAGAATTC** TTATTCAAGTTGGAAATAGGCTGTTTTGTC |
| pET6xHN-N-LY-NSP11 | LY-NSP11 | Forward | **GATAAGGCCTCTGTCGAC** GGGAGCTGTATGCCACTAC |
|  |  | Reverse | **AAGCGGCCGCCAGAATTC** TTATTCCAACTGGAAATAGGCGG |
| pET6xHN-N-VR-mNSP11 | VR-mNSP11 | Forward | **GATAAGGCCTCTGTCGAC** GGGTCGAGCTCTCCGCTC |
|  |  | Reverse | **AGGTGACATG** ***GGC*** **GCATCCT** CCAACGGTAG |
|  |  | Forward | **AGGATGC** ***GCC* CATGTCACCT** CCAGATACCTCCC |
|  |  | Reverse | **AAGCGGCCGCCAGAATTC** TTATTCAAGTTGGAAATAGGC *GGC* TTTG |
| pET6xHN-N-LY-mNSP11 | LY-mNSP11 | Forward | **GATAAGGCCTCTGTCGAC** GGGAGCTGTATGCCACTAC |
|  |  | Reverse | **ATGTAATGTG** ***GGC*** **ACACCCC** CCAACCGTG |
|  |  | Forward | **GGGGTGT *GCC*** **CACATTACAT** CAAAATACCTACC |
|  |  | Reverse | **AAGCGGCCGCCAGAATTC** TTATTCCAACTGGAAATAGGC *GGC* GGC |
| FUW-DsRed | DsRed | Forward | **CCGCGGCCCCGAATTC** ATGGACAACACCGAGGACG |
|  |  | Reverse | **GCTTGATATCGAATTC** TGGGAGCCGGAGTGGCG |
| FUW-DsRed-NSP11 | DsRed | Forward | **CCGCGGCCCCGAATTC** ATGGACAACACCGAGGACG |
|  |  | Reverse | **TACAGCTCCCTCCTCCTCCC** TGGGAGCCGGAGTGGCG |
|  | NSP11 | Forward | **GGGAGGAGGAGGGAGCTGTA** TGCCACTACCG |
|  |  | Reverse | **GCTTGATATCGAATTC** TCATTCCAACTGGAAATAGGCGG |

Note: Overlapping sequences for cloning vector and multi-fragments are in bold. Restriction sites are underscored. Mutant codons are Italianized.

**Table S3. Primers for qRT-PCR**

| **qRT-PCR Primers Table** | | |
| --- | --- | --- |
| **Target** |  | **Sequence (5’ → 3’)** |
| PRRSV-  LY | Forward | AAGATGACATCCGGCACCAC |
|  | Reverse | CCGGCAGCATAAACTCAACCTG |
| PRRSV-  VR | Forward | AAACCAGTCCAGAGGCAAGG |
|  | Reverse | GCAAACTAAACTCCACAGTGTAA |
| PRRSV-  NADC30 | Forward | GGATGGCCAGCCAGTCAATC |
|  | Reverse | TGACGTCATCTTCAGTCGCTAGAG |
| PRRSV-  SDSU73 | Forward | CCCTAGTGAGCGGCAATTGTGTC |
|  | Reverse | GGCGCACAGTATGATGCGTC |
| PRRSV-  SD16 | Forward | AAACCAGTCCAGAGGCAAGG |
|  | Reverse | GCAAACTAAACTCCACAGTGTAA |
| pGAPDH | Forward | CATCCTGGGCTACACTGAGG |
|  | Reverse | GCTTGACGAAGTGGTCGTTG |
| pIFN-β | Forward | TGCAACCACCACAATTCC |
|  | Reverse | CTGAGAATGCCGAAGATCTZ |
| IBV | Forward | AAGCGGTGTAGGTCATGGTG |
|  | Reverse | ACTTAGCAAGCCACTGACCC |
| ckGAPDH | Forward | GCAACCGTGTTGTGGACTTG |
|  | Reverse | GGGAACAGAACTGGCCTCTC |
